# Supplementary material for: Male foraging efficiency, but not male problem-solving performance, influences female mating preferences in zebra finches
Source: PeerJ. 2016 Aug 31;4:e2409. doi: 10.7717/peerj.2409 (PMC5012330; doi:10.7717/peerj.2409)
Supplement: Supplemental Information 1 — The asterisk indicates whether the initially less-preferred male had a better learning performance (i.e., required less trials to reach the learning criterion) than the other stimulus male, when data on learning speed were available for both males. [file peerj-04-2409-s001.docx]

**Supporting information : Raw data**

The asterix indicates whether the initially less-preferred male had a better learning performance (*i.e*., required less trials to reach the learning criterion) than the other stimulus male, when data on learning speed were available for both males

| ID | Treatment | Percent of choosing time with the initially less-preferred male | | Percent of time in the choosing zone | | Learning speed (*i.e*., number of trials to reach the learning criterion) | | |
| --- | --- | --- | --- | --- | --- | --- | --- | --- |
|  | Main=1  Control=2 | Before observation | After observation | Before  observation | After observation | Less-preferred male | Preferred male | Femelle |
| 1 | 1 | 0.50 | 0.50 | 1.00 | 1.00 | 53 | 66 | 42 |
| 2 | 1 | 0.41* |  | 0.78 |  | 66 | 53 | 37 |
| 3 | 1 | 0.39* | 0.58 | 0.9 | 0.90 | 98 | 24 | 28 |
| 4 | 1 | 0.18 | 0.67 | 0.54 | 0.97 | 24 | 98 | 35 |
| 5 | 1 | 0.46* | 0.32 | 1.00 | 0.99 | 66 | 53 | 15 |
| 6 | 1 | 0.23 | 0.59 | 0.73 | 0.06 | 24 | 98 | 30 |
| 7 | 1 | 0.28 | 0.69 | 0.92 | 0.57 | 24 | 98 | 65 |
| 8 | 1 | 0.13 | 0.60 | 0.98 | 0.97 | 53 | 66 | 88 |
| 9 | 1 | 0.04* | 0.95 | 0.95 | 0.89 | 98 | 24 | 7 |
| 10 | 1 | 0.32* | 0.77 | 0.93 | 0.99 | 66 | 53 | 58 |
| 11 | 1 | 0.28 | 0.13 | 0.80 | 0.98 | 24 | 98 | 34 |
| 12 | 1 | 0.00* | 0.70 | 1.00 | 0.88 | 98 | 24 | 48 |
| 13 | 1 | 0.47 | 0.84 | 0.53 | 0.31 | 53 | 66 | 39 |
| 14 | 1 | 0.47* | 1.00 | 0.92 | 1.00 | 98 | 24 | 62 |
| 15 | 1 | 0.45 | 0.08 | 0.16 | 0.96 | 53 | 66 | 40 |
| 16 | 1 | 0.47* | 0.13 | 0.73 | 0.46 | 66 | 53 | 74 |
| 17 | 1 | 0.47 | 0.36 | 0.71 | 0.37 | 24 | 98 | 41 |
| 18 | 1 | 0.20 | 0.99 | 0.94 | 0.55 | 53 | 66 | 15 |
| 19 | 2 | 0.44 | 0.68 | 0.99 | 0.98 |  |  | 42 |
| 20 | 2 | 0.49 | 0.62 | 0.96 | 0.94 | 18 | 66 | 28 |
| 21 | 2 | 0.18 | 0.02 | 0.93 | 0.99 | 7 | 64 |  |
| 22 | 2 | 0.46 | 0.39 | 0.89 | 0.97 |  |  | 41 |
| 23 | 2 | 0.50 | 0.49 | 0.92 | 0.87 |  |  | 7 |
| 24 | 2 | 0.50 | 0.50 | 0.98 | 0.97 | 18 | 66 | 37 |
| 25 | 2 | 0.50 | 0.54 | 0.99 | 1.00 | 64 | 7 | 48 |
| 26 | 2 | 0.45 | 0.57 | 0.99 | 0.34 |  |  | 74 |
| 27 | 2 | 0.28 | 0.59 | 0.88 | 0.95 |  |  | 40 |
| 28 | 2 | 0.45 | 0.86 | 0.93 | 0.95 | 18 | 66 | 50 |
| 29 | 2 | 0.27* | 0.50 | 0.99 | 0.99 | 64 | 7 | 34 |
| 30 | 2 | 0.32 | 0.50 | 0.73 | 0.98 |  |  | 65 |
